# The effects of awe on interpersonal forgiveness: the mediating role of small-self

**DOI:** 10.3389/fpsyg.2024.1336068

**Published:** 2024-02-06

**Authors:** Suxia Liao, Yichang Liu, Bo Yuan

**Affiliations:** Department of Psychology, Ningbo University, Ningbo, Zhejiang, China

**Keywords:** awe, interpersonal forgiveness, small-self, economic game, computational modeling

## Abstract

Awe could increase prosocial behavior, but little is known about its effects on interpersonal forgiveness. This study aims to explore the potential impact of awe on interpersonal forgiveness and the underlying mechanism of this process, using a combination of questionnaires, economic game and computational modeling. In Study 1, we utilized Trait Awe Scale (TAS) and Forgiveness Trait Scale (FTS) to examine the association between trait awe and trait forgiveness. In Study 2, we employed pre-screened video to induce awe, happy and neutral emotions, then evaluated the effects of induced awe on small-self and interpersonal forgiveness in hypothetical interpersonal offensive situations (Study 2a) and two economic interaction situations (Study 2b). Results from Study 1 indicate that there is a positive correlation between trait awe and trait forgiveness. Study 2 reveal that awe can enhance interpersonal forgiveness in both interpersonal conflict situations and economic interaction situations, and this effect is mediated by the sense of small-self elicited by awe. Overall, these findings contribute to our understanding of the potential impact of awe on interpersonal forgiveness and provide valuable insights into the mechanisms through which awe may influence forgiveness. Further research in this area could help to elucidate the potential applications of awe-based interventions in promoting forgiveness and positive social interactions.

## 1 Introduction

Social relationship is a fundamental aspect of human life. However, conflicts and disharmony can occur in even the closest relationships, which can result in emotional, social, material, and physical harm (McCauley et al., 2022). Forgiveness has been identified as an effective means of dealing with such conflicts. Research has shown that interpersonal forgiveness could reduce individual levels of anxiety, stress, and anger (Ryan and Kumar, 2005; van der Wal et al., 2016), promote prosocial motivation (Milburn, 2015), and decrease the likelihood of aggressive behavior (Quintana-Orts and Rey, 2018). Therefore, it is meaningful to explore the ways of encouraging forgiveness and reconciliation following conflicts or disharmony.

Interpersonal forgiveness is a process in which the victim's negative perceptions, emotions, and behavioral responses to the offender turn into positive ones after a conflict (Enright, 1991). According to McCullough et al. (1998), forgiveness is a series of motivational changes that encourage the victim to empathize with the offender, which reduces victim's tendency for retaliation and alienation toward the offender and strengthens the motivation to reconcile with them. Worthington and Wade (1999) suggested that forgiveness involves removal of negative emotions toward the offender and expression of positive emotions. The essence of interpersonal forgiveness is a prosocial change toward the offender, despite their hurtful actions, which includes downregulation of negative emotions toward the offender (Karremans et al., 2020).

Research has consistently shown that interpersonal forgiveness arises from a synergy between an individual's inherent personality traits and external environment in which they live (Koutsos et al., 2008). For instance, some studies suggest that an individual's self-esteem has an impact on forgiveness after an individual feels offended (Yao et al., 2017); compared with individuals with low self-esteem, those with high self-esteem are more likely to forgive others. Individuals with high agreeableness tend to have internal control of attribution, which will also increase their level of forgiveness (Eaton et al., 2006). As regards the external environment, a previous study suggests that offenders' intent and the severity of the consequences of the offense significantly influence the level of forgiveness by the victim; unintentional and minor offenses are more easily forgiven by the offended (Witvliet et al., 2020).

While previous research has unraveled a variety of facilitators of forgiveness, one constant barrier is the victim's absorption in the emotional aftermath of the offense (Karremans et al., 2020). When people are insulted or injured, they might get highly occupied with their hurt sentiments. Thoughts and emotions can be experienced as an integral part of the self (Bernstein et al., 2015) and produce a state known as subjective realism, in which the content of one's thoughts and emotions are experienced as reality rather than reflected upon with metacognitive awareness (Lebois et al., 2015; Papies et al., 2015). When an individual closely identifies with the present experiences of being harmed, he or she may be immersed in negative emotion and less likely to take the offender's perspective, which may impede forgiveness.

According to research on psychological distance, transcending an egocentric viewpoint helps people see the big picture, think holistically and abstractly, and make sense of and perceive meaning from their life events (Trope and Liberman, 2010; Kross and Ayduk, 2011; Waytz et al., 2015). A few studies have found that people who experience awe tend to consider the broad picture rather than concrete details, attending to abstract, high-construal-level information rather than concrete details. Valdesolo and Graham (2014) suggest that awe may enhance individual cognitive flexibility, making individuals more receptive to new experiences, which assist people in organizing and integrating life experiences across situations and time, and explain why and how the events occurred (Habermas and Bluck, 2000; McLean, 2008; McLean et al., 2010). This kind of thinking can help individuals reexamine the offending event from a more objective perspective, thus reducing the negative emotions of being offended and increasing the possibility of interpersonal forgiveness.

Awe becomes a complex emotional response, elicited when individuals confront phenomena that transcend their existing frameworks of comprehension. This multifaceted emotion envelops feelings ranging from admiration and wonder to confusion and surprise (Keltner and Haidt, 1999, 2003). Even though certain awe experiences can carry undertones of trepidation, awe is predominantly construed as a positive emotion (Keltner and Haidt, 2003; Bonner and Friedman, 2011). Awe, considered a self-transcendent positive emotion, can bolster cognitive processes and enhance cognitive flexibility. They also facilitate sharing during interpersonal exchanges (Bagozzi et al., 1999), and augment the capacity for positive cognitive appraisals (Isen, 2001).

Existing studies suggest that awe is associated with prosocial behavior. For instance, studies have shown that individuals with high trait awe are more likely to experience humility (Stellar et al., 2018), are more inclined to donate more money in economic games, show more generosity, and display helpful behavior (Piff et al., 2015; Prade and Saroglou, 2016). Induced awe also increases individual's sense of belonging and connection to the group and generates altruistic motivation (Shiota et al., 2007). In addition, awe also could reduce aggressive behavior (Ying et al., 2016) and antisocial behavior (Bai et al., 2017).

Prevailing explanation for why awe has prosocial effects is that awe reduces self-focus and produces a metaphorically small self (Perlin and Li, 2020). The concept of a small-self, introduced by Keltner and colleagues, involves a reduction in self-awareness, self-focus, egocentricity, and a diminishing emphasis on self-relevance (Keltner and Haidt, 2003). Research has consistently shown that awe can reduce self-focus and produce a metaphorical small-self (Shiota et al., 2007; Campos et al., 2013; Piff et al., 2015; Bai et al., 2017). Tyson et al. (2022) suggested that small-self is not as a singular, momentary state, but a rich, multidimensional construal that might endure even in the absence of immediate stimuli. According to previous literature, small-self is mainly composed of two structures: self-size and self-focus. Self-size, a measure introduced by Bai et al. (2017), relates to individuals' perceptions of feeling small and insignificant, particularly when experiencing awe (Shiota et al., 2007). Self-focus, on the other hand, was conceptualized by Piff et al. (2015). This construct highlights how experiencing awe can shift attention away from the self toward larger entities and collective aspects of personal identity. Together, these constructs provide a comprehensive understanding of how awe-inspiring experiences can lead to a sense of a smaller, less significant self, and redirect focus from individualistic to more collective dimensions of existence.

Some studies show that small-self has been associated with a range of positive outcomes, including helping behavior (Piff et al., 2015), egalitarianism (Hornsey et al., 2018), and collective engagement (Bai et al., 2017). For example, Campbell et al. (2004) found that people who experience small-self are more likely to give more money to groups and display more helpful actions in interpersonal interactions. The notion that a small self is a central characterization of the experience of awe, and that it is an active ingredient in promoting prosocial responses to awe, is referred to as the “small-self hypothesis” (Bai et al., 2017).

Regarding the effect of awe on the act of forgiveness, we speculate that the sense of small-self evoked by awe may prompt individuals to view the offending event in a broader perspective by reducing their focus on self. Previous studies have suggested that creating psychological distance from a negative event when thinking about it could produce adaptive consequences and facilitate forgiveness (Rizvi and Bobocel, 2016). Thus, awe allows people to turn their attention away from themselves and see the world in a humble way, helping them become more open and inclusive and also fosters forgiveness behavior in them. Although previous studies have analyzed the influencing factors of interpersonal forgiveness from many aspects, no research has explored the effect of awe on interpersonal forgiveness, which plays an important role in promoting interpersonal relationships in social interactions and cultivating healthy personalities (McCullough et al., 1998).

Therefore, the present study aims to examine the association between awe and interpersonal forgiveness, as well as the potential mediating role of small-self in this relationship. To achieve this, a combination of questionnaires, economic games, and computational modeling will be used. In Study 1, we use the Trait Awesome Scale (TAS) and Forgiveness Trait Scale (TFS) to investigate the association between trait awe and trait interpersonal forgiveness. In Study 2a and 2b, we employ pre-screened videos to induce the emotion of awe, explore subsequent impact on interpersonal forgiveness in a hypothetical interpersonal offensive situation and economic exchange situation, and tentatively examine the potential mediation effect of small-self.

## 2 Study 1: relationship between the trait of awe and the trait of interpersonal forgiveness

### 2.1 Participants

We performed a pre-estimated statistical power analysis for correlational analysis using the R *pwr* package (Champely, 2018), which indicated that a minimum sample size of 84 participants was necessary to detect a significant effect (α = 0.05; 1−β = 0.80) of correlation with a medium effect size (*r* = 0.30) (Cohen, 1992). A total of 180 Chinese undergraduate or graduate students were recruited from an online survey platform. Four participants were excluded from data analyses as they did not respond to all questions in the questionnaire. Thus final data was obtained from 176 participants, including 118 female participants, who had a mean age of 21.29 (*SD* = 2.74).

### 2.2 Measurements

#### 2.2.1 Trait awe scale

Trait awe was assessed using the Trait Awe Scale (TAS), a subscale of the Dispositional Positive Emotion Scales (DPES), which has been widely used in previous studies to measure positive emotion dispositions (Shiota et al., 2007). The trait awe scale was translated by Dong (2016), a Chinese researcher in 2016, and the findings of a confirmatory factor analysis showed that factor loading of item 1 was under 0.40. The TAS therefore has five items after localization, item 1 was eliminated from the scale (e.g., “I have many opportunities to see beautiful nature”), and participants rated their agreement to each statement on a 7-point scale (*1* = *totally disagree; 7* = *totally agree*). The sum of agreement ratings to the five items was used as an index of trait awe (Cronbach's α = 0.78), with a higher sum score indicating a higher level of trait awe.

#### 2.2.2 Forgiveness trait scale

McCullough et al. (2003) divided forgiveness into trait forgiveness and situational forgiveness. Trait forgiveness is a relatively stable individual tendency, which means that individuals show the same tendency to forgive under different offending situations. Situational forgiveness means that individuals show different forgiveness behaviors under different offending situations. Studies have proved that there is a separation between trait forgiveness and situational forgiveness (Brose et al., 2005). Therefore, this study explores the relationship between awe and interpersonal forgiveness from the perspectives of trait forgiveness and situational forgiveness.

The trait forgiveness was measured using the Forgiveness Trait Scale (FTS) developed by Berry et al. (2001). The FTS consists of 10 statements (e.g., “I am someone who forgives others easily”), and participants rated their agreement with each statement on a 5-point scale (*1* = *totally disagree; 5* = *totally agree*). We calculated the average of the ratings as a composite score for the trait of interpersonal forgiveness (Cronbach's α = 0.75). A higher score indicates a greater tendency to forgive others.

### 2.3 Statistical approach

We fitted a general linear model to explore the relationship between awe and interpersonal forgiveness after controlling for other possible confounding factors (e.g., gender and age). Apart from classical approach in frequentist framework, a Bayesian linear model was also used to estimate the relationship between two variables, which were performed in R (R Core Team, 2020) with brms (Bürkner, 2017) and RStan (Stan Development Team, 2018).

### 2.4 Results

**Figure 1:**
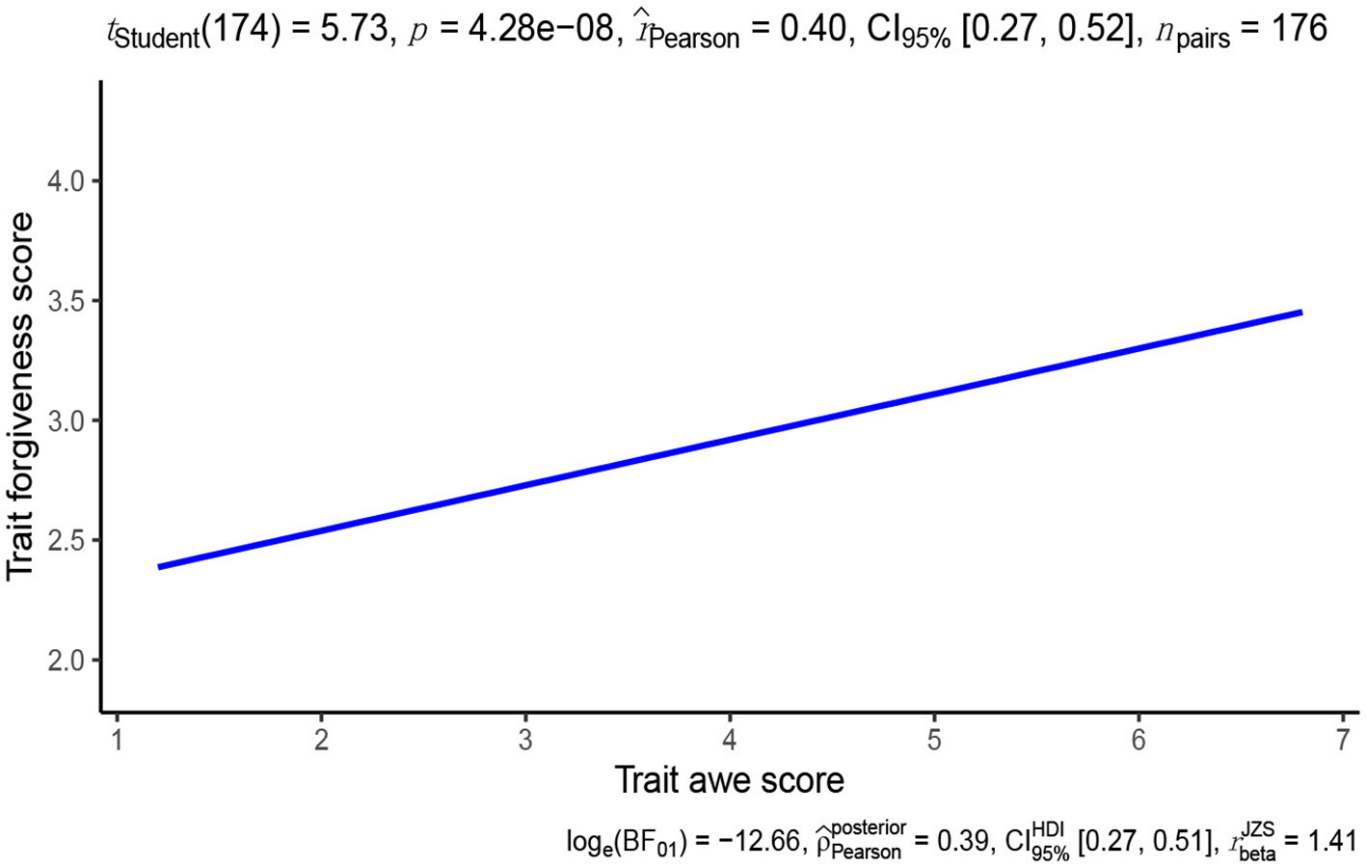
Scatter plot depicting the relationship between trait awe and interpersonal forgiveness.

According to Curran et al. (1996), skewness >2 and kurtosis >7 indicate a severe deviation from the normal distribution. The distribution of trait of awe and interpersonal forgiveness was negatively skewed (*skewness* = −1.02, *kurtosis* = 4.66; *skewness* = −0.01, *kurtosis* = 2.73), but basically fit the normal distribution. There was a significant positive relationship between the trait of awe and the trait of interpersonal forgiveness, *r*_(174)_ = 0.40, 95% *CI* = [0.27, 0.52], *p* < 0.001, indicating that the higher level of trait awe, the higher level of interpersonal forgiveness (see Figure 1).

A linear regression analysis was conducted to assess the relationship between trait awe and trait interpersonal forgiveness. The results showed a significant positive association (β = 0.19, 95% *CI* [0.12, 0.26], *t* = 5.73, *p* < 0.001), indicating that for every one-point increase in trait awe score, there was a corresponding increase of 0.19 points in trait interpersonal forgiveness on a 5-point scale. This positive effect remained significant even after controlling for potential confounding variables such as gender, age, and religion or non-religion (β = 0.17, 95% *CI* [0.11, 0.24], *t* = 5.32, *p* < 0.001) (see Table 1). Furthermore, the Bayesian linear model also provided a similar regression estimate (β = 0.17, *SE* = 0.03, 95% *CIs* [0.11, 0.24]).

**Table 1:** Estimated coefficients from the linear regression analysis in Study 1.

**Predictor**	**β**	***SE***	***t***	***p***
Intercept	1.99	0.34	5.96	< 0.001
Trait awe	0.17	0.03	5.32	< 0.001
Age	0.01	0.01	0.45	0.654
Gender	0.14	0.07	2.05	0.042
Religion or not	0.51	0.18	2.77	0.001

### 2.5 Discussion

In Study 1, a questionnaire was used for examining the differences in trait awe across various demographic variables. The results revealed that trait awe did not show significant differences based on age, while significant differences were observed in relation to religion and gender. More importantly, the results of Study 1 show that there is a significant positive correlation between individual trait awe and trait forgiveness. Although there is no direct evidence to suggest that awe can increase people's interpersonal forgiveness behavior, existing research has found that awe increases prosocial behavior in individuals (Piff et al., 2015; Ying et al., 2016). Interpersonal forgiveness is seen as a prosocial behavior, and it makes sense that awe can boost levels of interpersonal forgiveness.

## 3 Study 2: the influence of induced awe on interpersonal forgiveness

The finding from Study 1 suggests a positive association between the trait of awe and the trait of interpersonal forgiveness, which provides preliminary support for our hypothesis. However, a causal relationship between awe and forgiveness cannot be reliably established. Thus, Studies 2a and 2b were conducted to examine whether induction of awe would lead to increased interpersonal forgiveness compared to a neutral or happy emotion induction, and to explore the potential mediating role of small-self in this relationship. These experimental studies aim to provide evidence for a causal impact of awe on interpersonal forgiveness and shed light on the underlying psychological mechanism.

### 3.1 Study 2a: the influence of induced awe on forgiveness in the interpersonal offensive situation

#### 3.1.1 Participants

We performed a pre-estimated statistical power analysis for one-way analysis of variance using the R *pwr* package (Champely, 2018). A between-subjects design was employed with three conditions: awe, happy, and neutral. Based on an effect size (*f* = 0.30), type I error rate α = 0.05, and statistical power 1−β = 0.80, the R *pwr* package required a minimum of 37 participants per cell. To ensure adequate power, we aimed at 40 participants per cell. A total of 135 participants (84 females; mean age = 20.44, *SD* = 2.34) were recruited and randomly assigned to one of the three conditions: awe (*n* = 45), happy (*n* = 45), and neutral (*n* = 45).

#### 3.1.2 Materials and methods

##### 3.1.2.1 The awe manipulation (priming) task

The pre-screened videos were used to induce the emotion of being in awe, happy, and neutral. All video clips were taken from Bai et al. (2017), and each video had a duration of about 2 min. The awe of nature video was chosen from panoramic views of nature from the BBC's *Planet Earth* series, which included panoramic views of beautiful landscapes such as waterfalls, deserts, oceans, large rivers, and high mountains. The happy video was excerpted from well-known humor series, Mr. Bean. The neutral video was excerpted from a short documentary, *A Bite of China*, depicting how to make pickles (see Supplementary material for more information).

We conducted a pretest to ensure that each video was effective in inducing the respective target emotion. After watching 2-min video respective to their condition, participants completed an emotional assessment scale to make sure the videos effectively induced the expected emotion. The Emotional Assessment Scale includes 7 items (happy, awe, fear, sadness, anger, gratitude, aversion), all using a 7-point Likert scale, ranging from 1 (*totally disagree*) to 7 (*totally agree*).

Furthermore, we also used the Awe Component Assessment Scale to verify the successful induction of awe emotion. This questionnaire consists of three items: “(1) the situation makes me feel vast,” “(2) I feel challenged about my worldview,” and “(3) I see a different world.” The last two items were combined to assess the need for accommodation (Cronbach's α = 0.92), which is scored on a 7-point scale (1 = *completely disagree*, 7 = *completely agree*).

##### 3.1.2.2 The small-self assessment

The small-self assessment consists of self-size scale and self-focus scale. The scores of two scales were calculated separately and added together. The lower scores, the stronger the sense of small-self which was induced by awe.

**Figure 2:**
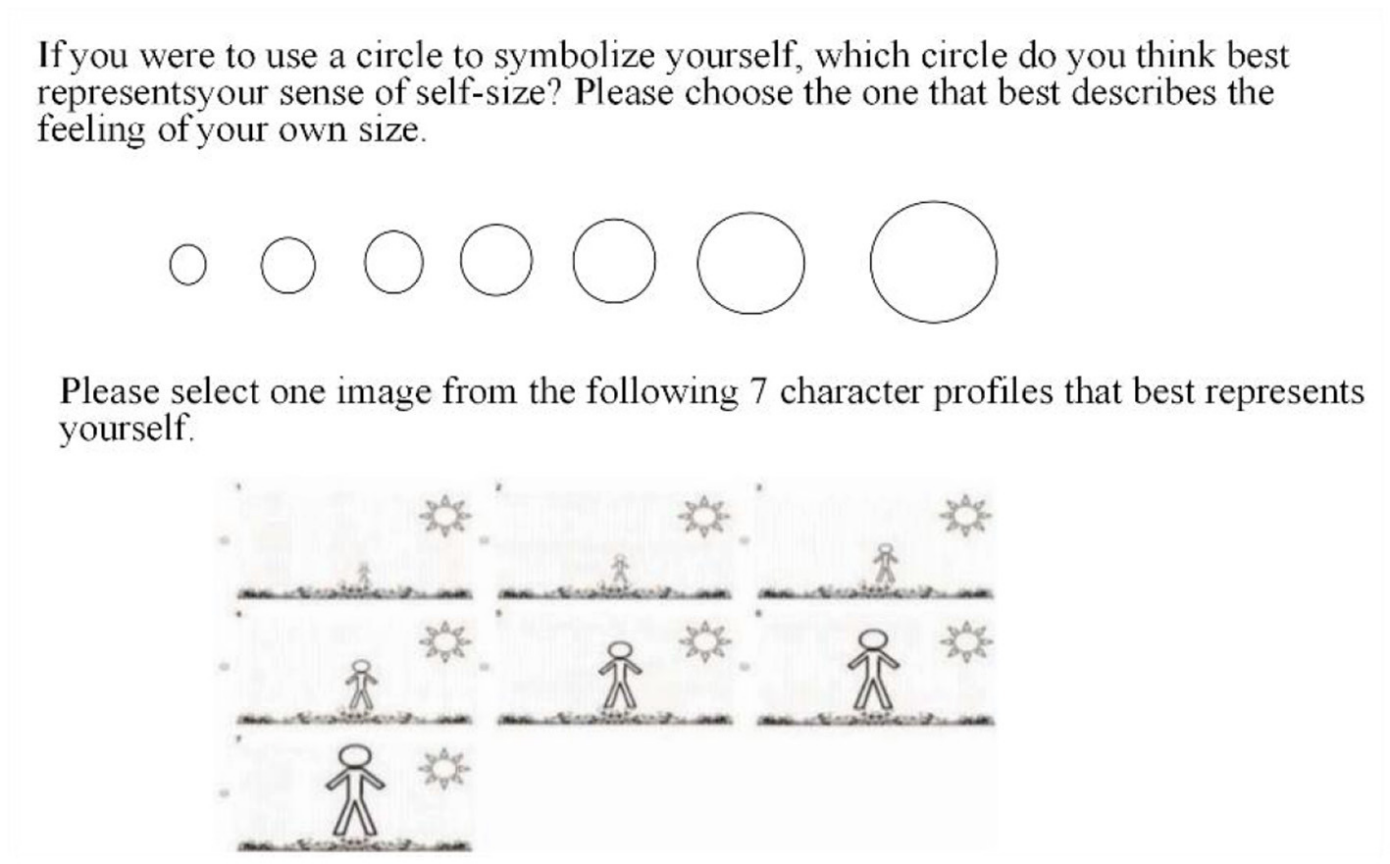
Illustration of self-size scale in Study 2a.

The self-size scale includes five topics (e.g., *compared to scene in video, I feel quite small*), of which the first two items are scored in reverse and the others are self-aware image measurement (Bai et al., 2017). Specifically, participants were provided with a series of seven circles, full-body images and signatures, from which they were asked to choose the one that best represent their perceived size (see Figure 2). Participants rated their agreement to each of the five statements on a 7-point scale, ranging from 1 (*totally disagree*) to 7 (*totally agree*) to indicate their perception of self-size (Cronbach's α = 0.78).

The self-focus scale was used to measure the tendency for the individual toward self-focus (Woody, 1996), which is a subscale of the Focus of Attention Questionnaire and consists of five items. Participants were instructed to focus on their current state and respond with their agreement from 1 (*strongly disagree*) to 5 (*strongly agree*) to items such as “*I focus on what I would say or do next*” or “*I am concerned about past social failures*” (Cronbach's α = 0.77).

##### 3.1.2.3 The interpersonal forgiveness assessment

Interpersonal forgiveness was measured by inducing a hypothetical interpersonal offensive situation in daily life. Participants were asked to imagine themselves as protagonists of the situational scenario. The specific situation is: “*Li is one of your classmates. Without your permission, he/she secretly took your computer for his/her brother's use for up to a month. During this period, you have asked him/her, but he/she didn't tell you the truth, and said he/she didn't know. For this you delayed a lot of tasks assigned by teacher because you didn't have a computer, and your teacher punished you for this*.” After reading the scenario, participants completed a Chinese version of the Transgression-Related Interpersonal Motivations Inventory (TRIM) to measure their interpersonal forgiveness in a hypothetical interpersonal offensive situation.

The TRIM was developed by McCullough et al. (1998), which consists of 12 items assessing revenge (e.g., “*When I think about the incident, I wish that something bad would happen to him/her*”) and avoidance (e.g., “*When I think about the incident, I would rather avoid him/her*”) toward the offender. All items were scored on a 5-point scale, ranging from 1 (*completely disagree*) to 5 (*completely agree*). After reverse scoring revenge and avoidance items, we averaged the items and, as in previous research (Campbell et al., 2004; Santelli et al., 2009), refer to this total score as a level of interpersonal forgiveness (Cronbach's α = 0.88).

##### 3.1.2.4 Procedure

In experiment 2a, participants were initially randomly assigned to one of the three conditions (awe, happy, or neutral) to induce specific emotion states. After watching the video, participants completed the small-self assessment scale. Following the video watching and self-assessment, they read a hypothetical interpersonal offensive situational scenario and imagine themselves as the protagonist. Finally, they completed the TRIM scale to assess their level of interpersonal forgiveness.

#### 3.1.3 Results

First, a one-way ANOVA was conducted to compare the ratings of sense of awe and happy between the three emotion conditions. The results showed a significant difference among three conditions for both sense of awe [*F*_(2, 132)_ = 74.67, *p* < 0.001, η^2^ = 0.53] and happy [*F*_(2, 132)_ = 134.75, *p* < 0.001, η^2^ = 0.67]. Specifically, participants in awe condition reported higher sense of awe (*M* = 5.91, *SD* = 1.33) than those in the happy condition [(*M* = 3.40, *SD* = 1.16), *t*_(132)_ = 9.53, *p* < 0.001, *d* = 2.01], and in neutral condition [(*M* = 2.91, *SD* = 1.26), *t*_(132)_ = 11.39, *p* < 0.001, *d* = 2.40]. There was no significant difference between happy and neutral condition [*t*_(132)_ = 1.86, *p* = 0.066, *d* = 0.39]. Participants in the happy condition reported a higher level of happy (*M* = 6.16, *SD* = 0.71) than those in the awe condition [(*M* = 4.84, *SD* = 0.74), *t*_(132)_ = 8.14, *p* < 0.001, *d* = 1.72] and in the neutral condition [(*M* = 3.51, *SD* = 0.84), *t*_(132)_ = 16.42, *p* < 0.001, *d* = 3.46]. The participants in awe condition reported a higher level of happy (*M* = 4.84, *SD* = 0.74) than those in the neutral condition [(*M* = 3.51, *SD* = 0.84), *t*_(132)_ = 8.28, *p* < 0.001, *d* = 1.74]. These results indicate that the manipulation of awe and happy emotions was successful.

To compare the ratings of The Awe Component Assessment Scale between three emotion conditions, a one-way ANOVA was performed. The results showed a significant difference among three conditions for the sense of awe [*F*_(2, 132)_ = 68.06, *p* < 0.001, η^2^ = 0.49]. Participants in the awe condition reported a higher sense of awe (*M* = 14.96, *SD* = 3.83) than those in happy condition [(*M* = 10.71, *SD* = 3.09), *t*_(132)_ = 6.16, *p* < 0.001] and in neutral condition [(*M* = 6.92, *SD* = 3.17), *t*_(132)_ = 11.66, *p* < 0.001]. Participants in the neutral condition reported a lower sense of awe (*M* = 6.92, *SD* = 3.17) than in happy condition [(*M* = 10.71, *SD* = 3.09), *t*_(132)_ = −5.50, *p* < 0.001]. These results indicate that the manipulation of awe emotions was successful.

Only a few participants in our study reported that they were religious, and almost all the participants were non-religious. We did not the compare differences in interpersonal forgiveness behavior between them. Through the independent sample *t* test, on the differences in college students' trait awe between gender, the only child status on interpersonal forgiveness was analyzed. The results indicated a significant differences between gender, and the boys were found to exhibit a lower level of situational interpersonal forgiveness (*M* = 36.94, *SD* = 3.57) compared to girls [(*M* = 39.26, *SD* = 3.62), *t*_(132)_ = −3.28, *p* = 0.001] and there are no differences in the only child status [*t*_(132)_ = −0.81, *p* = 0.419]. Students who are the only child report a higher score on interpersonal forgiveness (*M* = 39.19, *SD* = 4.56) than those who are not the only child (*M* = 38.58, *SD* = 3.47). Consequently, it is necessary to include gender as a covariate in subsequent studies.

**Figure 3:**
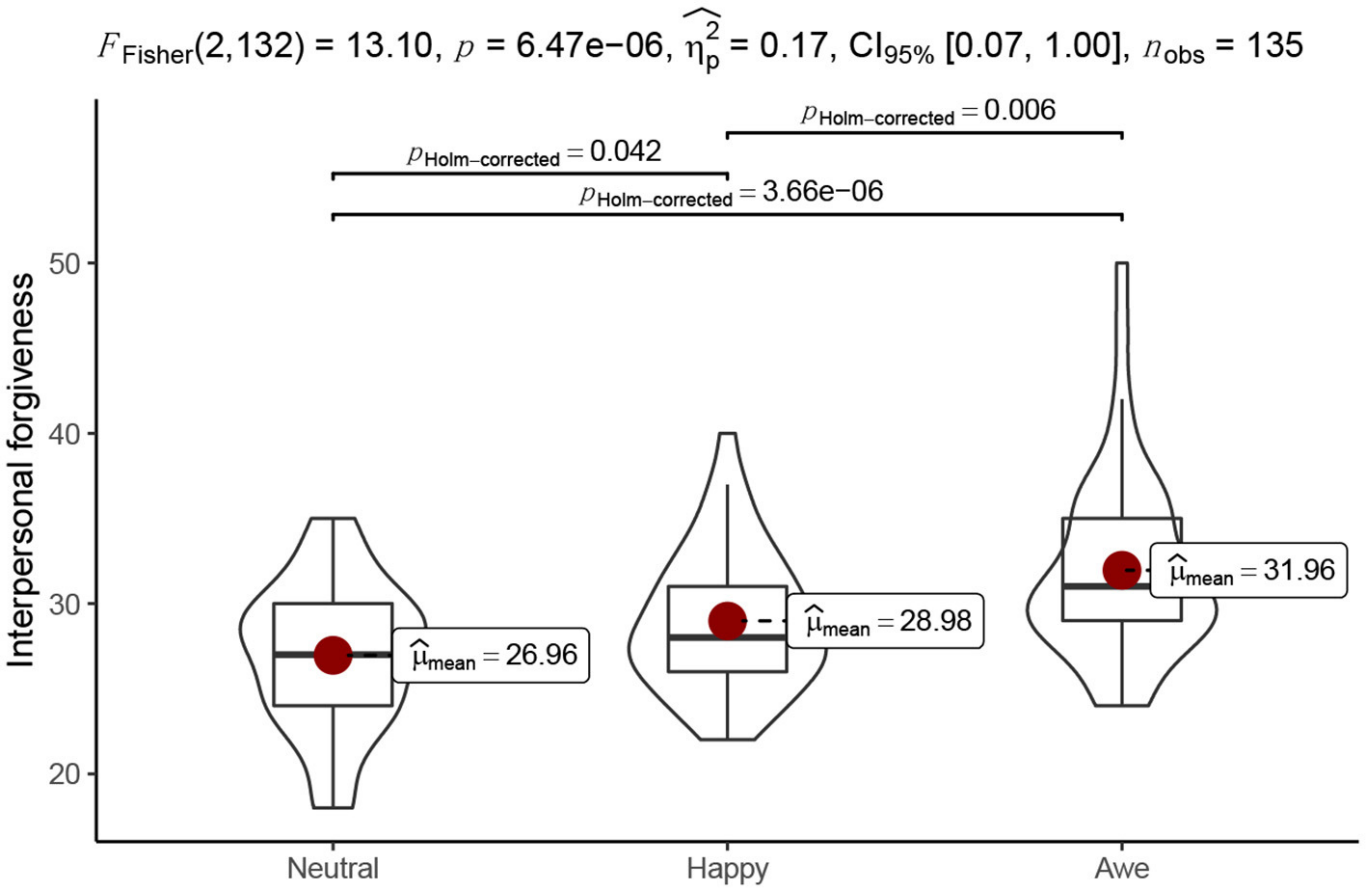
Interpersonal forgiveness levels under different emotional conditions.

Next, we conducted an analysis to investigate the effect of induced awe on interpersonal forgiveness in the hypothetical interpersonal offensive situation. The index of interpersonal forgiveness was the reversed total score of retaliation and avoidance motivation. The results revealed a significant difference between three emotion conditions on interpersonal forgiveness [*F*_(2, 118)_ = 13.10, *p* < 0.001, η^2^ = 0.17]. Specifically, participants in awe condition show higher interpersonal forgiveness (*M* = 31.96, *SD* = 5.47) than those in happy condition [(*M* = 28.98, *SD* = 4.10), *t*_(132)_ = 3.03, *p* = 0.004, *d* = 0.64] and in neutral condition [(*M* = 26.96, *SD* = 4.30), *t*_(132)_ = 5.09, *p* < 0.001, *d* = 1.07]. In addition, there was a significant difference between happy and neutral conditions, [*t*_(118)_ = 2.06, *p* = 0.042, *d* = 0.43] (see Figure 3).

**Figure 4:**
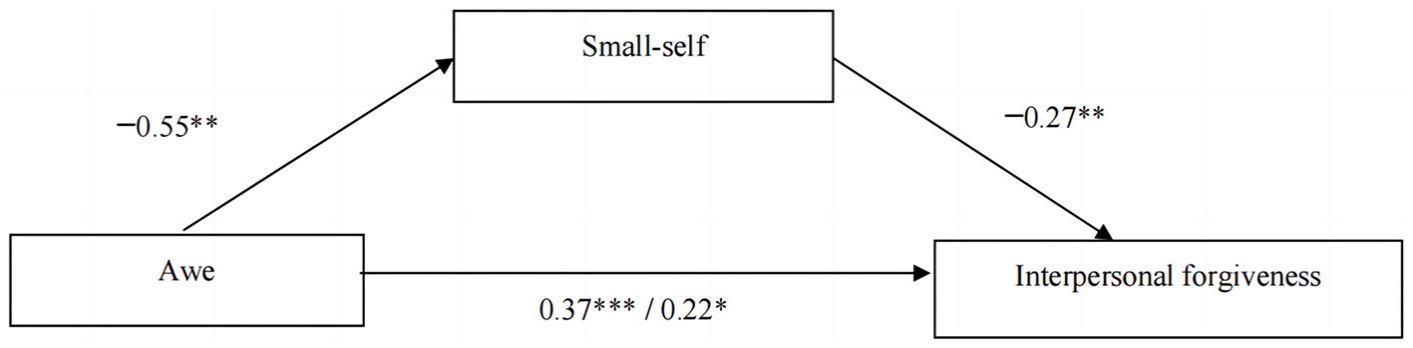
Mediation effect of small-self between awe and interpersonal forgiveness in the hypothetical interpersonal offensive situation (**p < 0.01, ***p < 0.001).

Next, we conducted a mediation analysis to examine the role of small-self in the relationship between induced awe and interpersonal forgiveness. We used a bias-corrected bootstrapping method with 5,000 resamples, as recommended by Preacher and Hayes (2004). The results showed that the mediating effect of small-self was significant (β = 0.15, 95%*CI* [0.02, 0.31]) (see Figure 4). The findings suggest that the effect of induced awe on interpersonal forgiveness is partially mediated by a sense of small-self evoked by awe.

**Figure 5:**
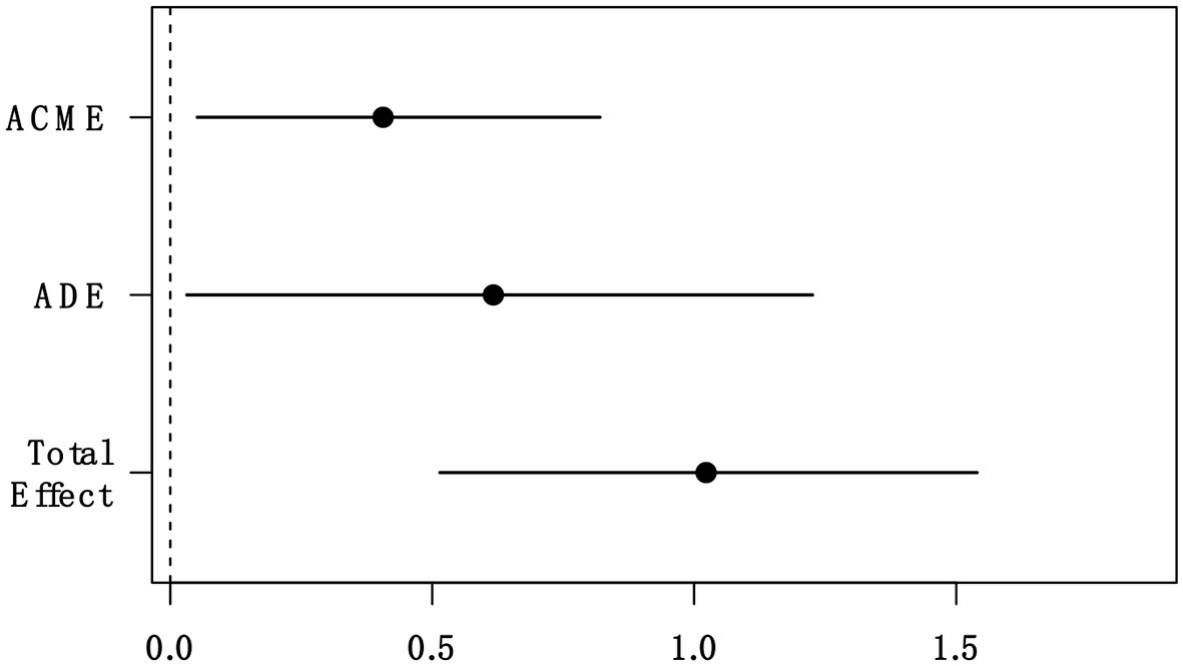
The mediation effect of small-self. ACME, average causal mediation effect; ADE, average direct effect.

We also examined the mediation effect of small-self using the R package, mediate (Tingley et al., 2014). This package uses a model-based inference approach to estimate the average causal mediation effect (ACME, i.e., indirect effect), average direct effect (ADE), and the average total effect. We used 1,000 bootstrap re-samplings and quasi-Bayesian approximated bias-corrected and accelerated confidence intervals. For each mediation model, two regressions were fitted: the mediator model and the outcome model. The mediator model regressed the mediator on the independent variable, the outcome model regressed bonding on the independent variable and mediator. The mediation effect of small-self was significant (ACME = 0.41 [0.51, 0.82], *p* = 0.018). The effect size of this mediation effect was large (40.45%). Figure 5 depicts the results of the mediation models.

#### 3.1.4 Discussion

The results show that awe can increase the level of interpersonal forgiveness, and small-self evoked by awe plays a mediating role in it. Awe prompts individuals to turn their attention to the external environment, reduces their perception of self-importance, and places them into a larger frame of reference (Van Cappellen and Saroglou, 2012), which can weaken self-awareness and reduce self-concern. When individuals are less concerned about their own interests, the retaliatory behavior of actively maintaining their own interest will be correspondingly reduced (Ying et al., 2016).

### 3.2 Study 2b: the influence of induced awe on forgiveness in the economic exchange situation

#### 3.2.1 Participants

The power analysis procedure for Study 2b was the same as that in Study 2a. A total of 120 Chinese undergraduate or graduate students (73 female participants) were recruited from an online survey platform. One participant was excluded from data analyses due to unforeseen interruption of the program, resulting a final data from 119 participants, with a mean age of 20.36 (*SD* = 2.31). Participants were randomly assigned to one of the three conditions: awe (*n* = 40), happy (*n* = 39), or neutral (*n* = 40).

#### 3.2.2 Materials and methods

##### 3.2.2.1 The awe manipulation (priming) task

This part was consistent with Study 2a.

##### 3.2.2.2 The small-self assessment

This part was consistent with Study 2a.

##### 3.2.2.3 The interpersonal forgiveness assessment

In Study 2b, Ultimatum Game (UG) and Prisoner's Dilemma Game (PDG) were used to assess interpersonal forgiveness in an economic interaction situation. In the Ultimatum Game, unfair offers often elicit a provocative response from the recipient (Prasad et al., 2017). The multi-round one-shot UG offers several advantages over other commonly used measures of aggression. Notably, the decisions made in UG are less susceptible to social desirability bias, which can affect self-reported aggression tendencies (Krumpal, 2013). Additionally, multi-round one-shot UG effectively reduces the potential impact of long-term strategic considerations, where participants repeatedly play against the same individual, thus allowing for a more accurate assessment of immediate responses (Cueva et al., 2017). Moreover, recent studies have introduced computational models that can be applied to UG, providing a solid foundation for exploring the intricate psychological mechanisms underlying aggressive behavior (Xiang et al., 2013; Gu et al., 2015). These advancements contribute to a more comprehensive understanding of aggression through the lens of UG.

Prisoner's Dilemma Game provides an exceptional context for studying forgiveness for two essential reasons (Wallace et al., 2008). Firstly, it captures ubiquitous trade-off between self-interest and cooperation that exists in real-life situations. Secondly, extensive research has shown that when confronted with non-cooperation in social dilemma studies, individuals often experience anger (Dawes et al., 1977) and frequently employ a tit-for-tat strategy, ultimately mirroring non-cooperative behavior of their counterpart (Kelley and Stahelski, 1970; Kuhlman and Marshello, 1975; Van Lange and Visser, 1999). Consequently, non-cooperative behavior in social dilemmas tends to evoke a desire for revenge, which directly contradicts the concept of forgiveness (McCullough et al., 1998). The inclination of individuals to react with anger and adopt retaliatory strategies in the face of non-cooperation underscores the challenge of cultivating forgiveness in such scenarios.

Overall, multi-round one-shot UG and PDG serves as valuable tools for examining interpersonal forgiveness, offering improved validity and less susceptibility to biases compared to other measures. By utilizing computational models, researchers can delve deeper into psychological factors contributing to interpersonal forgiveness within the context of UG and PDG.

##### 3.2.2.4 Procedure

In the experiment, participants were randomly assigned to one of three conditions to induce awe, happy, or neutral emotions, respectively. After watching a video, participants filled in a small-self assessment scale. Then they completed UG and PDG, which were presented using the PsychoPy software (Peirce, 2009). The whole experiment lasted for about 35 min (See Supplementary material for a detailed description of the UG and GPD).

#### 3.2.3 Statistical approach

To examine the potential psychological mechanisms underlying the effects of awe on interpersonal forgiveness, we conducted a model-based analysis based on the participants' refusal response in UG. Computational model fitting allowed us to test whether the sense of awe influences individuals' sensitivity to provocation and norm adaptation rates (Detailed computational modeling is provided in Supplementary material).

#### 3.2.4 Results

As in Study 2a, we first conducted emotion manipulation check. There was a significant difference between three emotion conditions on awe rating in emotion priming task [*F*_(2, 116)_ = 67.71, *p* < 0.001, η^2^ = 0.54]. Participants in the awe condition reported a greater sense of awe (*M* = 5.93, *SD* = 1.37) than those in the happy condition [(*M* = 3.31, *SD* = 1.22), *t*_(118)_ = 9.13, *p* < 0.001, *d* = 2.05] and in the neutral condition [(*M* = 2.85, *SD* = 1.23), *t*_(118)_ = 10.80, *p* < 0.001, *d* = 2.42], but there was no significant difference between happy and neutral conditions [*t*_(118)_ = 1.60, *p* = 0.113, *d* = 0.36]. In addition, there was a significant difference between different emotion condition on happy rating [*F*_(2, 118)_ = 131.76, *p* < 0.001, η^2^ = 0.69]. Participants in the happy condition reported greater happy emotion (*M* = 6.23, *SD* = 0.68) than those in the awe condition [(*M* = 4.80, *SD* = 0.76), *t*_(118)_ = 8.45, *p* < 0.001, *d* = 1.88) and in the neutral condition [(*M* = 3.45, *SD* = 0.85), *t*_(118)_ = 7.99, *p* < 0.001, *d* = 1.79]. Participants in the awe condition reported greater happy emotion (*M* = 4.80, *SD* = 0.76) than those in the neutral condition [(*M* = 3.45, *SD* = 0.85), *t*_(118)_ = 7.96, *p* < 0.001, *d* = 1.78], indicating successful manipulation of awe and happy emotions.

Next, we examined whether the induced awe could influence interpersonal forgiveness in UG. There was a significant difference between the three emotion conditions on rejection rate in the UG task [*F*_(2, 118)_ = 30.70, *p* < 0.001]. Participants in the awe condition displayed a lower rejection rate (*M* = 0.40, *SD* = 0.11) than those in the happy condition [(*M* = 0.59, *SD* = 0.10), *t*_(116)_ = −7.64, *p* < 0.001, *d* = −1.72] and in the neutral condition [(*M* = 0.53, *SD* = 0.12), *t*_(116)_ = −5.29, *p* < 0.001, *d* = −1.18]. In addition, participants in the happy condition (*M* = 0.59, *SD* = 0.10) show a higher rejection rate than those in the neutral condition [(*M* = 0.53, *SD* = 0.12), *t*_(116)_ = 2.38, *p* = 0.019, *d* = 0.54]. In addition, the results showed that compared to happy and neutral conditions, the awe condition decreased disgust rating [*F*_(2, 116)_ = 6.62, *p* = 0.002, η^2^ = 0.100] but did not have a significant impact on happy [*F*_(2, 116)_ = 0.23, *p* = 0.794, η^2^ = 0.004], anger [*F*_(2, 116)_ = 1.67, *p* = 0.192, η^2^ = 0.028], and disappointment [*F*_(2, 116)_ = 1.27, *p* = 0.285, η^2^ = 0.021] ratings.

**Figure 6:**
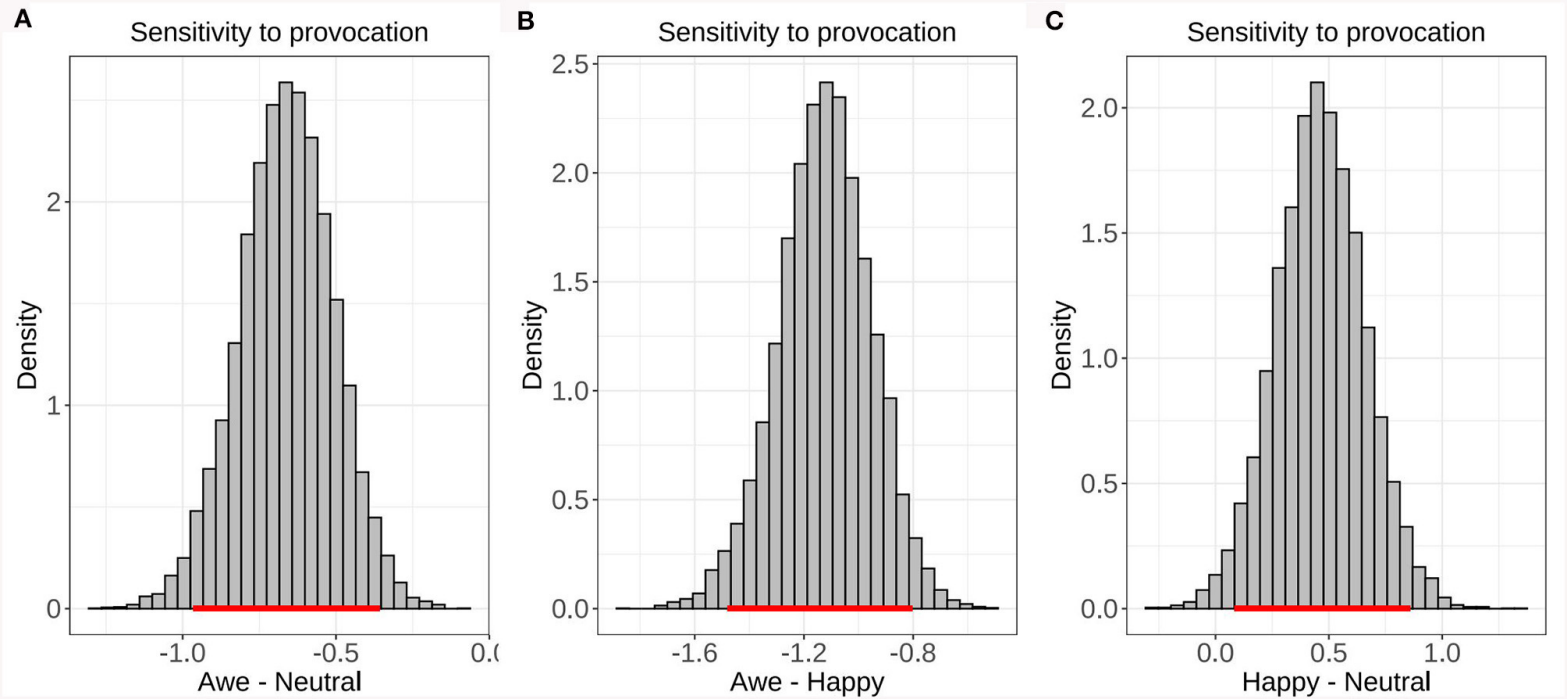
The posterior distributions of the difference in the sensitivity to provocation parameters α between the awe happy and neutral conditions. The red line indicates the 95% HDI. The effect is significant if the red line does not overlap zero. **(A)** Illustrate that there is a significant difference in sensitivity to provocation between Awe and Nertral. **(B)** Illustrate that there is a significant difference in sensitivity to provocation between Awe and Happy. **(C)** Illustrate that there is a significant difference in sensitivity to provocation between Happy and Nertral.

Furthermore, we conducted a model-based analysis to examine the potential psychological mechanisms underlying the effects of awe on interpersonal forgiveness in the UG. Compared with the happy and neutral emotions, awe significantly decreased the sensitivity to provocation parameters α (the 95% highest density interval [HDI] of the posterior distribution of α_*awe*_−α_*neutral*_). Compared with happy and neutral, awe significantly decreased the sensitivity to provocation parameters α (the 95% highest density interval [HDI] of the posterior distribution of α_*awe*_-α_*neutral*_: [−0.97, −0.36], α_*awe*_-α_*happy*_: [−1.48, −0.80], α_*happy*_-α_*neutral*_: [0.08, 0.86]) (see Figure 6). Compared with happy and neutral emotions, awe had no significant effect on the norm adaption rate parameters ε or the inverse temperature parameter τ (see the Supplementary Figures S1, S2).

**Figure 7:**
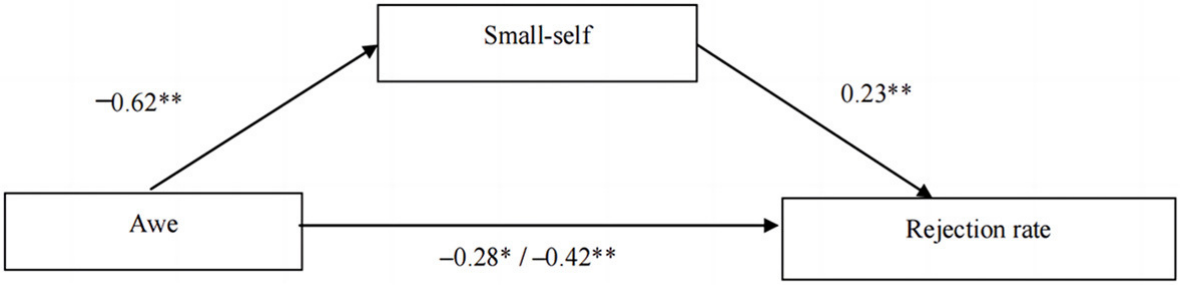
Mediation effect of small-self between awe and interpersonal forgiveness in the hypothetical interpersonal offensive situation (*p < 0.05, **p < 0.01).

A mediation analysis was conducted to test the indirect effects of induced awe on rejection rate through the small-self. The results, based on a bootstrapping method with 5,000 resamples, indicated a significant mediating effect of small-self (β = −0.14, 95%*CI* [−0.30, −0.001]). The analysis showed that induced awe significantly reduced rejection rate in the UG, and that this effect was partially mediated by small-self. A graphical representation of the mediation model is presented in Figure 7.

**Figure 8:**
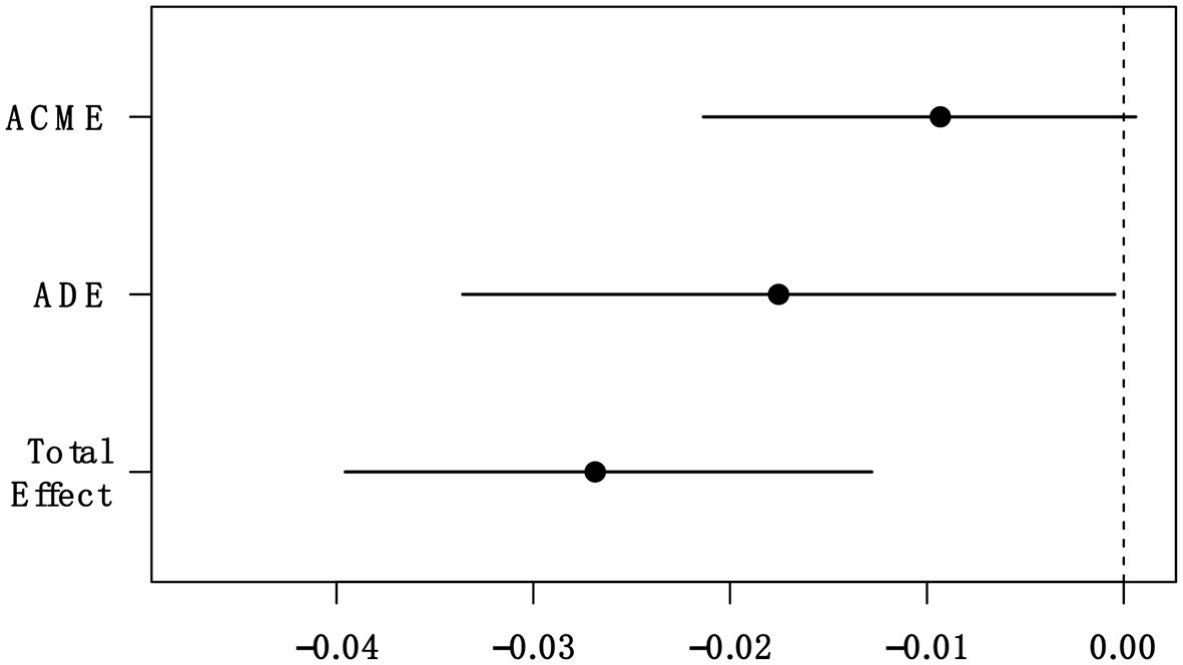
The mediation effect of small-self. ACME, average causal mediation effect; ADE, average direct effect.

Similar to Study 2a, we also examined the mediation effect of small-self using the R package, mediate, and the same parameter settings (Tingley et al., 2014). The mediation effect of small-self was significant (ACME = −0.01 [−0.02, −0.001], *p* = 0.068) (Figure 8). The effect size of this mediation effect was 33.96%. The findings suggest that the effect of induced awe on interpersonal forgiveness is partially mediated by a decrease in small-self evoked by awe.

We also analyzed whether there was a difference in the proportion of participants choosing cooperation in the third round of the PDG task across the three emotional conditions. First, a chi-square test was performed to compare the ratio of participants selecting cooperation under these three conditions. The results showed that there was a significant difference among three groups [$\chi_{\left(2\right)}^{2}$ = 22.77, *p* < 0.001]. We conducted a Mann–Whitney *U* test for the three groups, and the results revealed that participants in the awe condition exhibited a higher cooperation rate (*M* = 0.70, *SD* = 0.46) compared to those in the happy condition [(*M* = 0.21, *SD* = 0.41), *U* = 1,166.00, *p* < 0.001], as well as compared to those in the neutral condition [(*M* = 0.30, *SD* = 0.46), *U* = 1,120.00, *p* < 0.001]. Furthermore, there was no significant difference between the happy (*M* = 0.21, *SD* = 0.41) and neutral conditions [(*M* = 0.30, *SD* = 0.46), *U* = 854.00, *p* = 0.0339]. Secondly, we used evoked emotion as a predictive variable to conduct logistics regression analysis for binary cooperative choice (1, 0). The results showed that compared with the neutral emotion, awe increased cooperative behavior (β = 1.69, *SE* = 0.49, *p* < 0.001) and there was no difference between happy and neutral emotions (β = −0.51, *SE* = 0.53, *p* = 0.335).

#### 3.2.5 Discussion

The results of Study 2 shed light on unique impact of awe emotions, particularly the small-self associated with awe. These findings are consistent with previous research (Griskevicius et al., 2010; Rudd et al., 2012; Van Cappellen and Saroglou, 2012; Ying et al., 2016). In addition, participants who experienced awe reported feeling a significantly small-self compared to those experiencing neutral and happy emotions, which is consistent with the observations of Bai et al. (2017). Experiment 1 and partial results from experiment 2 provide evidence that small-self plays a mediating role in the effect of awe on interpersonal forgiveness behavior, supporting the concept of small-self.

To further investigate other potential mechanisms linking awe and interpersonal forgiveness, a computational modeling analysis based on ultimatum game was conducted. Three potential psychological processes were examined: victim sensitivity, adaptability to social norms, and emotion. The results show that compared to happy and neutral emotions, awe may decrease offender sensitivity and increase adaptability to social norms. Previous studies have shown a significant negative correlation between offender sensitivity and interpersonal forgiveness, suggesting that higher sensitivity to victimization leads to a lower likelihood of forgiveness (Strelan and Sutton, 2011). Therefore, the experience of awe may influence interpersonal forgiveness behavior by reducing victim sensitivity. Researchers consider awe to be a positive emotion that positively affects an individual's cognitive frame of reference, behavior, and action tendencies (Shiota et al., 2007); besides, awe may influence the extent of interpersonal forgiveness by enhancing an individual's ability to conform to social norms. In addition to these effects, awe may attenuate negative emotions experienced during the ultimatum game, particularly disgust.

## 4 Discussion

The current study aimed to investigate the association between awe and interpersonal forgiveness, as well as to explore the underlying mechanism of this association. In Study 1, we employed the Trait Awe Scale and the Trait Forgiveness Scale to examine the relationship between awe and interpersonal forgiveness. The results reveal that trait awe positively relates to the tendency to forgive. In Study 2, we used pre-screened videos to induce awe emotions and combined a hypothetical interpersonal offensive situation and two economic games to investigate the effects of induced awe on interpersonal forgiveness, as well as the underlying mechanism of this process. The results indicated that induced awe leads to greater individual interpersonal forgiveness behavior compared to happy or neutral emotions, and this effect was mediated by small-self evoked by awe.

Taken together, using a multimethod approach, we found a positive relationship between trait awe and interpersonal forgiveness, and observed that induced awe significantly increased interpersonal forgiveness. Our study advances the understanding of the positive effects of awe and its underlying psychological mechanism. Previous research has shown that awe reduces aggression, increases prosocial behavior, and a sense of small-self relative to neutral and happy emotions (Ying et al., 2016). Rankin and colleagues discovered that feeling awe, an expansive state of wonder and reverence, can help people successfully manage a challenging waiting period by enhancing patience and wellbeing while broadening people's perspectives (Rankin et al., 2020). The sense of small-self was found to mediate the effect of awe on aggression and prosocial behavior.

While previous research has extensively studied the effect of awe on prosocial behavior, there is a lack of corresponding research on whether awe can enhance interpersonal forgiveness. Interpersonal forgiveness is defined as a prosocial change toward the offender despite their hurtful acts, involving a transformation of the victim's attitudes and motivations toward the offender, with reduced tendencies to seek revenge or avoid the offender, and increased feelings of benevolence (McCullough et al., 1998). This transformation requires victims to undergo a process of working through the experience, suggesting that some form of cognitive engagement has taken place.

In the current study, we found that awe reduced individuals' avoidance and revenge motivation after interpersonal offense (Study 2a), decreased sensitivity to provocation, and increased cooperative behavior after being unfairly treated or betrayed in two economic interaction games (Study 2b). These results suggest that awe can indeed promote changes in motivation toward offenders, which in turn enhances interpersonal forgiveness. Based on our theoretical reasoning and previous empirical findings, we hypothesized that the association between awe and interpersonal forgiveness would be explained by a sense of small-self evoked by awe. Experiences of awe evoke a sense of small-self, shifting attention away from self toward others and the greater community (Piff et al., 2015). Previous research has shown that awe leads people to feel connected with others (Bai et al., 2017) and display prosocial behavior (Rudd et al., 2012; Piff et al., 2015; Prade and Saroglou, 2016; Stellar et al., 2018). Extending this line of research, we found that small-self evoked by awe appears to increase interpersonal forgiveness.

## 5 Limitations and future directions

It is important to acknowledge several limitations of the current research. This study uses natural landscape as material to elicit awe emotion. Based on the definition of awe, there are many sources of awe in addition to the grand scenes of nature, such as admirable deeds and small but exquisite things. In future, other triggers can be used to investigate whether awe can promote interpersonal forgiveness behavior. In addition, this study used video tasks in the laboratory to evoke awe in subjects. Although the evoked materials can elicit the feeling of awe effectively, considering the ecological validity, the VR technology or on-site research can also be used to elicit the feeling of awe so that participants can fully experience the feeling of awe.

As regards interpersonal forgiveness, in this study, ultimatum game and prisoner's dilemma game are combined as a research paradigm of interpersonal forgiveness behavior, and the rejection rate and cooperation rate in two tasks are respectively used as indicators of willingness for interpersonal forgiveness. However, since the operational definition of forgiveness is not clear, the measurement indicators of this experimental paradigm still need to be considered. In addition, hypothetical scenarios and economic game paradigms differ from real-life events, and further consideration is needed to fully extrapolate the research findings to real-world scenarios.

Since our findings were restricted to Chinese college students, it will be important to further study the effects of awe in non-student samples or people from other non-collectivist cultures in the West. While the research presented in this paper focuses on awe as a positive emotion, further work must be done to extend these findings to negative states of awe, such as fear-based awe experienced during, for example, thunderstorms, floods, and famines. This warrants careful examination in future studies.

## 6 Conclusion

In this study, we aimed to broaden our understanding of conditions that facilitate forgiveness by investigating the relationship between trait awe and the inclination to forgive others, as well as the effect of induced awe on interpersonal forgiveness. Using a multimethod approach, we demonstrated a positive association between trait awe and interpersonal forgiveness. We found that induced awe significantly enhanced the tendency to forgive and small-self evoked by awe appears to increase interpersonal forgiveness. Our findings replicate and extend previous research on awe as a distinct positive emotion and have important practical implications for conflict resolution in interpersonal relationships.

## Data availability statement

The data described in this article are openly available in the Open Science Framework at https://osf.io/n8sfy/.

## Ethics statement

The studies involving humans were approved by Ethics Committee of Ningbo University. The studies were conducted in accordance with the local legislation and institutional requirements. The participants provided their written informed consent to participate in this study.

## Author contributions

SL: Writing – original draft, Writing – review & editing. YL: Writing – original draft, Writing – review & editing. BY: Writing – original draft, Writing – review & editing.
