# Supplementary material for: The effects of awe on interpersonal forgiveness: the mediating role of small-self
Source: Front Psychol. 2024 Feb 6;15:1336068. doi: 10.3389/fpsyg.2024.1336068 (PMC10877021; doi:10.3389/fpsyg.2024.1336068)
Supplement: Supplementary file 1 [file Data_Sheet_1.docx]

**Supplementary Information for**

**The Effects of Awe on Interpersonal Forgiveness: The Mediating Role of Self-Focus**

### 2.3 Materials and Methods

As a multifaceted emotion, awe can be triggered by various sources, with natural wonders, remarkable achievements, and art being the most common inducers (Keltner & Haidt, 2003). In laboratory settings, researchers often use tasks involving videos, reading materials, or recall tasks to induce awe. One popular method is the video task, where participants are shown carefully curated clips to evoke feelings of awe (Saroglou et al., 2008; Rudd et al., 2012; Van Cappellen & Saroglou, 2012). For example, in a study conducted by Van Cappellen and Saroglou (2012), participants were divided into two groups. One group watched the BBC documentary "The Earth" to induce awe through the depiction of breathtaking natural landscapes, while the other group viewed videos of pregnant women giving birth, aiming to evoke awe towards the miracle of life. Awe-inducing situations can be further classified into social, physical, and cognitive triggers, with natural landscapes and human-made art being commonly employed (Keltner & Haidt, 2003; Piff et al., 2015).

To identify suitable research materials for inducing awe in Chinese college students, we designed a self-developed open-ended awe questionnaire based on existing research and interviews from Schneider's "Awakening Awe: A Personal Legend of Profound Transformation". The questionnaire consisted of four items, employing a content response approach. The specific questions were as follows: "Describe the feeling of awe (list two or three points)," "What do you think caused the feeling of awe (anything you can imagine)," "Recall one or two situations in which you experienced awe and describe them in as much detail as possible," and "How did the feeling of awe affect you?" The responses to the four questions provided by the participants were compiled, categorized, and summarized. The results are presented in Table 1.

**Table S1.** Statistics Table of Content of Open Questionnaire

|  | Classification (report frequency) | | | | |
| --- | --- | --- | --- | --- | --- |
| Understanding of awe | Respect、fear  （54） | Love、Admiration（63） | Sacred  （30） | Vast、Sense of distance（63） | others（14） |
| Awe-inducing cause | Physical incentives（90） | Social incentives（40） | Abstract  （22） | others  （5） |  |
| Awe-inducing scene | Natural scene  （78） | Related to people（63） | Abstract  （2） | others  （0） |  |
| The effect of awe | Cognition  （20） | emotion  （45） | Behavior  （88） | Ideas  （36） | others（0） |

Table 1 reveals that specific triggers can be classified into physical, social, and abstract situations. Among these, natural landscapes were mentioned more frequently in relation to physical situations compared to other categories, while abstract sources were mentioned the least. This demonstrates that nature, as a specific trigger, is capable of inducing awe emotions in a broader range of individuals.

Based on our investigation results, we compared and selected video content used in previous studies to induce awe, happy, and neutral emotions. We ultimately chose three videos used by Bai et al. (2017), each approximately two minutes in length, to induce awe, happy, and neutrality, respectively.

#### *3.2.2 Materials and Methods*

***the Ultimatum Game.*** The Ultimatum Game (UG) is a task that assesses behavioral responses to social provocation (Wang et al., 2011). The game involves two players, a proposer and a responder. The proposer offers a division of an endowment (e.g., ¥20, approximately $2.9) between themselves and the responder. If the responder accepts the offer, each player gets the proposed amounts. However, if the responder rejects the offer, neither player receives any money. Responders often view unfair offers as social provocation and are likely to reject them (Anderson & Bushman, 2002; Prasad et al., 2017).

In the UG task, participants played as the responder in 60 trials and were paid according to their decisions in three randomly selected trials. Participants were informed that they were interacting with a new anonymous player on each trial and that each proposal offer was from a different player who had previously visited the laboratory and had their offer recorded. However, unbeknownst to participants, the proposal offers came from two different Gaussian distributions. In the first and third blocks (20 trials per block), the offers to participants were generated from a Gaussian distribution with a mean of ¥8 and a standard deviation of ¥1.5. In the second block, the offers were generated from a Gaussian distribution with a mean of ¥4 and a standard deviation of ¥1.5 (Zhu et al., 2019). This manipulation was designed to change participants’ expectation about the offers across trials and facilitate a norm adaptation process. Additionally, considering that the repeatability of emotional assessment causes physiological and psychological response fatigue of the subjects, and the emotional state is unlikely to change greatly between adjacent rounds, we chose to evaluate the emotional state of the subjects in 60% of trials, participants rated their emotions in response to the offers they received after making their decision on a scale from 1 (very unpleasant) to 9 (very pleasant). After completing 60 UG trials, three kinds of emotions (anger, disappointment, and disgust) in response to previous proposals were measured on a 9-point scale (1 = very low, 9 = very high). The rejection rate of the offers was used as an indicator of reversed interpersonal forgiveness.

***the Prisoner's Dilemma Game.*** The Prisoner's Dilemma Game (PDG) is a paradigm that confronts individuals with a decision between short-term self-interest and long-term collective interests (Komorita & Parks, 1994). In this task, two players are presented with two options ('A' and 'B'). If both players choose 'A' (cooperation), both parties receive ¥60 (approximately $8.8) in this trial. If one player chooses 'A' and the other chooses 'B' (betrayal), the player who chooses 'A' receives ¥0 while the player who chooses 'B' receives ¥100 (approximately $14.7). If both players choose 'B', both parties receive ¥40 (approximately $5.9).

After completing the UG, participants played the PDG, which consisted of three rounds of decision-making. To establish expectation of future interactions, participants were informed that the task was a multi-round game, and if they want to win, they need follow a specific strategy. First, they must always choose 'A' (cooperate) before their partner chooses 'B' (betray); second, if the partner chooses 'B' first, they can also choose 'B'. In the three rounds of the game, the partner was set to always chooses to 'A' in the first round and 'B' in the second round. According to the instructions, in the third round, participants could choose to cooperate or betray freely based on their partner's betrayal in the second round. Therefore, participants’ choice in the third round indicates their reaction to the partner’s betrayal in the second round. After reading the instructions, participants answered four questions to ensure their understanding of the PDG task. If participants answered incorrectly, they were instructed to review the task instructions and answer the questions again until they understood them completely, after which they began the experiment.

**3.1.3 Results**

One-way ANOVA was conducted to compare the ratings of sense of self-size and self-focus between three emotion conditions. The results showed a significant difference among three conditions for both sense of self-size (*F*(2, 132) = 45.22, *p* < .001, η^2^ = 0.39) and self-focus (*F*(2, 132) = 5.59, *p* = .005, η^2^ = 0.07). Specifically, participants in awe condition reported lower sense of self-size (*M* = 3.50, *SD* = 1.08) than those in happy condition (*M* = 4.38, *SD* = 1.03), *t*(132) = −4.03, *p* < .001, *d* = −0.82, and in neutral condition (*M* = 5.56, *SD* = 1.08), *t*(132) = −9.47, *p* < .001, *d* = −1.93, participants in neutral condition reported higher sense of self-size (*M* = 5.56, *SD* = 1.08) than those in happy condition (*M* = 4.38, *SD* = 1.03), *t*(132) = 5.45, *p* < 0.001, *d* = 0.11. Participants in awe condition reported lower sense of self-focus (*M* = 23.08, *SD* = 2.84) than those in happy condition (*M* = 24.54, *SD* = 2.96), *t*(132) = −2.50, *p* = .036, *d* = −0.51, and in neutral condition (*M* = 24.93, *SD* = 2.79), *t*(132) = −3.18, *p* = .005, *d* = −0.65, there was no significant difference between happy(*M* = 24.54, *SD* = 2.96) and neutral condition (*M* = 24.93, *SD* = 2.79), *t*(132) = 0.68, *p* = .777, *d* = 0.14.

**Table S2**. Statistical results of emotional evaluation after different emotional priming in study 2a

|  | Awe video (*n*=88) | neutral video (*n*=88) | Happy video (*n*=88) | *F* | *p* |
| --- | --- | --- | --- | --- | --- |
| awe(*M±SD*) | 5.91*±*1.33 | 2.91*±*1.26 | 3.40*±*1.16 | 74.67 | < .001 |
| Happy(*M±SD*) | 4.84*±*0.74 | 3.51*±*0.84 | 6.16*±*0.71 | 134.75 | < .001 |
| sadness (*M±SD*) | 1.27±0.54 | 1.53±0.87 | 1.42±0.72 | 1.55 | 0.217 |
| anger (*M±SD*) | 1.33±0.48 | 1.64±0.91 | 1.31±0.70 | 3.04 | 0.051 |
| fear (*M±SD*) | 1.89±1.05 | 1.71±1.01 | 1.89±1.05 | 7.12 | < .001 |
| gratitude (*M±SD*) | 4.44±1.46 | 2.33±1.40 | 3.93±1.47 | 26.31 | <.001 |
| aversion (*M±SD*) | 1.31±0.97 | 2.78±1.46 | 1.49±0.87 | 22.56 | <.001 |

### 3.2.3 Statistical approach

**Table S3**. Statistical results of emotional evaluation after different emotional priming in study 2b

|  | Awe video (*n*=88) | neutral video (*n*=88) | Happy video (*n*=88) | *F* | *p* |
| --- | --- | --- | --- | --- | --- |
| awe(*M±SD*) | 5.93*±*1.34 | 2.85*±*1.23 | 3.31*±*1.22 | 67.71 | < .001 |
| Happy(*M±SD*) | 4.80*±*0.76 | 3.45*±*0.85 | 6.23*±*0.68 | 131.76 | < .001 |
| sadness (*M±SD*) | 1.88±0.97 | 1.78±1.05 | 1.15±0.66 | 2.31 | 0.104 |
| anger (*M±SD*) | 1.35±0.48 | 1.65±0.86 | 1.28±0.60 | 3.53 | 0.032 |
| fear (*M±SD*) | 1.88±0.97 | 1.78±1.05 | 1.15±0.66 | 7.49 | < .001 |
| gratitude (*M±SD*) | 4.50±1.52 | 2.15±1.25 | 3.75±1.43 | 29.24 | <.001 |
| aversion (*M±SD*) | 1.20±0.46 | 2.85±1.51 | 1.43±0.78 | 30.90 | <.001 |

**3.2.4 Results**

Computational model fitting allowed us to test whether sense of awe influence individuals’ sensitivity to provocation and norm adaptation rates. Specifically, we assumed that participant’s behavior can be modeled by individual aversion to unequal splits. The responder’s utility on iteration or round *i* of exchange can be represented using the Fehr–Schmidt (FS) inequality aversion utility as follows (Fehr & Schmidt 1999):

$$V_{i}\left( s_{i} \right)=s_{i}-\alpha\max\left\{ f_{i}-s_{i}, 0 \right\}$$

Here, α represents sensitivity to provocation (“envy”, $\alpha$ [0, 1]), $f_{i}$ is the offer a responder expects to receive (also called “internal norm”). That is, subject’s unwillingness to accept an offer $s_{i}$ below expected value $f_{i}$ (for details see, Gu et al. 2015). The larger parameter $\alpha$ is, the less likely a responder will accept an offer below their expectation. In addition, individuals update their expectations of offers $f_{i}$ as a function of previously received offers (Gu et al., 2015; Xiang et al., 2013). This means that perceived provocation (or unfairness) of same objective offer amount can change over time. To capture this effect, we used Rescorla-Wagner reinforcement algorithm (Rescorla & Wagner, 1972):

$$f_{i}=f_{i-1}-\varepsilon(s_{i}-f_{i-1})$$

where ε is adaption rate (or learning rate) ($\varepsilon$ ∈ [0, 1]), which represents the extent to which previous offer expectation ($f_{i-1}$) is updated by experienced offer $s_{i}$. The initial norm $f_{0}$ was fixed to be 10 (Zhu et al., 2019).

Given $V_{i}\left( s_{i} \right)$, we model the probability of accepting an offer as follows:

$$p_{i}\left( s_{i} \right)= \frac{e^{\tau V_{i}\left( s_{i} \right)}}{1+ e^{\tau V_{i}\left( s_{i} \right)}}$$

Here $\tau$ is the inverse temperature parameter of the softmax. The lower is $\tau$, the more diffuse and variable are the choices ($\tau$ [0, 10]). We use $\alpha$ parameter as an indicator of sensitivity to provocation and $\varepsilon$ parameter representing norm adaption rate.

Parameter estimation was conducted via the hBayesDM package by using data from all participants and from each condition (i.e., awe, happy, and neutral) (Ahn et al., 2017). In the hBayesDM package, posterior inference of the parameters was performed with a Markov Chain Monte Carlo technique implemented in the Stan (Carpenter et al., 2017). We drew 1000 samples from an initial burn-in step and 4000 new samples with four chains. Gelman-Rubin convergence tests were conducted for each parameter (Gelman & Rubin, 1992). All latent variables had 𝑅 < 1.05, which indicated all chains converged. The posterior highest density interval (HDI) represented the uncertainty in the estimated parameters. If the 95% HDI did not overlap zero, the effect was considered significant (Carpenter et al., 2017).

We conducted model-based analysis to examine potential psychological mechanisms underlying the effects of awe on interpersonal forgiveness in the UG. The results suggested that awe compared with happy and neutral had no significant effect on the norm adaption rate parameters $\varepsilon$ **(**see Figure S1**)** or the inverse temperature parameter $\tau$ **(**see Figure S2**)**.

Figure S1. The posterior distributions of the difference in the norm adaption rate parameters

Figure S2. The posterior distributions of the difference in the inverse temperature parameters

## References

Ahn, W. Y., Haines, N., & Zhang, L. (2017). Revealing neurocomputational mechanisms of reinforcement learning and decision-making with the hBayes DM package. *Computational Psychiatry*, *1*, 24–57. http://doi.org/10.1162/CPSY_a_00002

Anderson, C. A., & Bushman, B. J. (2002). Human aggression. *Annual Review of Psychology*, *53*(1), 27. https://doi.org/10.1146/annurev.psych.53.100901.135231

Carpenter, B., Gelman, A., Hoffman, M.D., Lee, D., Goodrich, B., Betancourt, M., et al. (2017). Stan: a probabilistic programming language. J. Stat. Softw. *76*. https://doi.org/10.18637/jss.v076.i01

Fehr, E., Schmidt, K.M. (1999). A theory of fairness, competition, and cooperation. *The Quarterly Journal of Economics*. *114*(3), 817–868. https://www.jstor.org/stable/2586885

Gu, X., Wang, X., Hula, A., Wang, S., Xu, S., Lohrenz, T. M., Montague, P. R. (2015). Necessary, Yet Dissociable Contributions of the Insular and Ventromedial Prefrontal Cortices to Norm Adaptation: Computational and Lesion Evidence in Humans. *Journal of Neuroscience*, *35*(2), 467–473. https://doi.org/10.1523/JNEUROSCI.2906-14.2015

Keltner, D., & Haidt, J. (2003). Approaching awe, a moral, spiritual, and aesthetic emotion. *Cognition & Emotion, 17*(2), 297–314. <https://doi.org/10.1080/02699930302297>

Piff, P. K., Dietze, P., Feinberg, M., Stancato, D. M., & Keltner, D. (2015). Awe, the small self, and prosocial behavior. *Journal of Personality and Social Psychology, 108*(6), 883–899. https://psycnet.apa.org/doi/10.1037/pspi0000018

Prasad, S., Narayanan, J., Lim, V.K.G., Koh, G.C.H., Koh, D.S.Q., Mehta, P.H. (2017). Preliminary evidence that acute stress moderates basal testosterone’s association with retaliatory behavior. *Hormones and Behavior*. *92*, 128–140. https://doi.org/10.1016/j.yhbeh.2016.10.020

Rudd, M., Vohs, K. D., & Aaker, J. (2012). Awe expands people’s perception of time, alters decision making, and enhances well-being. *Psychological Science*, *23*(10), 1130–1136. https://doi.org/10.1177/0956797612438731

Saroglou, V., Buxant, C., & Tilquin, J. (2008). Positive emotions as leading to religion and spirituality. *The Journal of Positive Psychology*, *3*(3), 165–173. https://doi.org/10.1080/17439760801998737

Van Cappellen, P., & Saroglou, V. (2012). Awe activates religious and spiritual feelings and behavioral intentions. *Psychology of Religion and Spirituality*, *4*(3), 223–236. <https://doi.org/10.1037/a0025986>

Xiang, T., Lohrenz, T., & Montague, P. R. (2013). Computational Substrates of Norms and Their Violations during Social Exchange. *Journal of Neuroscience*, *33*(3), 1099–1108. https://doi.org/10.1523/JNEUROSCI.1642-12.2013

Zhu, R., Liu, C., Li, T., Xu, Z., Fung, B., Feng, C., Wu, H., Luo, Y., & Wang, L. (2019). Intranasal oxytocin reduces reactive aggression in men but not in women: A computational approach. *Psychoneuroendocrinology*, *108*, 172–181. https://doi.org/10.1016/j.psyneuen.2019.06.016
